# Challenges and opportunities for incentivising antibiotic research and development in Europe

**DOI:** 10.1016/j.lanepe.2023.100705

**Published:** 2023-07-26

**Authors:** Michael Anderson, Dimitra Panteli, Robin van Kessel, Gunnar Ljungqvist, Francesca Colombo, Elias Mossialos

**Affiliations:** a Department of Health Policy, London School of Economics and Political Science, London, United Kingdom; b European Observatory on Health Systems and Policies, Brussels, Belgium; c Faculty of Health, Medicine and Life Sciences, Department of International Health, School CAPHRI (School for Public Health and Primary Care), Maastricht University, Maastricht, Netherlands; d Health Division, Organisation for Economic Co-operation and Development, Paris, France

**Keywords:** Antibiotic resistance, Antimicrobial resistance, Drug development

## Abstract

Antimicrobial, and particularly antibiotic resistance are one of the world’s biggest challenges today, and urgent action is needed to reinvigorate the antibiotic development pipeline. To inform policy discussions during and after the 2023 Swedish Presidency of the Council of the European Union, we critically appraise incentive options recently proposed by the European Commission, and member states, and consider what has been achieved over the last two decades in relation to antibiotic research and development. While several new antibiotics have achieved regulatory approval in recent years, almost none have innovative characteristics such as new chemical classes or novel mechanisms of action. We consider four incentive options to incentivise research and development of new antibiotics, including subscription payments, market entry rewards, transferable exclusivity extensions, and milestone payments. While each option has advantages and drawbacks, a combination of incentives may be required and continued investment is needed by the EU in push incentives, such as direct funding and grants, to incentivise drug discovery and preclinical stages of development. The EU must also coordinate with international initiatives and support access to new and pre-existing antibiotics in LMICs through platforms such as the WHO, and G7 and G20 group of countries.

## Introduction

Antimicrobial and particularly antibiotic resistance is one of the most significant challenges facing healthcare systems worldwide. In EU/EEA countries, antibiotic-resistant infections resulted in more than 38,000 deaths and more than 1 million years of life lost because of premature death or years lived with disabilities in 2019 alone.^1^ The economic impact of antimicrobial resistance (AMR) is also significant, costing the European Union (EU) approximately EUR 1.1 billion annually in additional healthcare costs.^2^ Drivers of AMR range from overuse of antimicrobials to poor infection prevention and control (IPC) and involve actors across the One Health spectrum, including human, animal, and environmental health.^3^ In addition, the WHO described the antibiotic pipeline as “insufficient” to meet growing AMR,^4^ and market failures for antibiotics mean few incentives for the pharmaceutical industry to invest in antibiotic research and development exist.^5^

The COVID-19 pandemic has re-emphasised that infectious diseases do not respect national borders, and there is renewed debate at the EU level on what can be done to tackle growing AMR, including planned revisions to general pharmaceutical legislation,^6^ the establishment of the Health Emergency Response Authority (HERA),^7^ and the development of the EU Global Health Strategy.^8^ The recently published Council of the European Union recommendations on AMR also include commitments to develop and implement incentives that strengthen research and development of new treatments for resistant infections, and access to pre-existing and new antimicrobials across member states.^9^ To inform discussions during and after the 2023 Swedish Presidency of the Council of the European Union, the purpose of this health policy article is to complement a report commissioned on this topic by the Swedish government by critically appraising four incentive options for antibiotic research and development proposed by the European Commission (transferable exclusivity extensions),^6^ and member states (subscription payments, market entry rewards, and milestone payments).^10^^,^^11^ In addition, we consider what has been achieved over the last two decades in relation to antibiotic research and development.

There are several important policy issues relevant to AMR that are outside the scope of this article. This article does not focus on policies necessary to limit the emergence and dissemination of resistant infections such as stewardship, IPC, public awareness, surveillance, and access to generic antibiotics.^12^, ^13^, ^14^ However, we acknowledge that investment in research and development of new antibiotics must be balanced with investment in conservation of pre-existing antibiotics, particularly as investment in IPC and stewardship is highly cost-effective and can significantly reduce the health burden of AMR.^14^ There are also many other incentive options for antibiotic research and development not considered in this article.^15^^,^^16^ These include, but are not limited to, the “pay or play” model,^17^ priority review vouchers,^18^ the options models for antibiotics (OMA),^19^ and models for public ownership of antibiotic intellectual property, and clinical trial networks.^20^ While these incentive options have not been recently proposed by the European Commission, or member states, they warrant further examination and should be the subject of additional research and analysis. While this article focuses on the European perspective, we also believe that policy options to secure sustainable access to new and pre-existing antibiotics in low and middle income countries (LMICs) warrants further research and analysis.

## What has been achieved over the last decade in relation to antibiotic research and development?

Undoubtedly, a significant success has been raising the profile of AMR among global and national policy makers, evidenced by the 2017 EU One Health Action Plan on AMR,^21^ the 2015 WHO Global Action Plan on AMR,^22^ and the 2016 UN declaration on AMR.^23^ This has kept incentivising antibiotic research and development high on the EU, WHO, G7, and G20 policy agenda. There have been several landmark reports, such as the 2015 Review on AMR,^24^ those produced for different presidencies of the European Council,^12^^,^^15^^,^^25^^,^^26^ and by DRIVE-AB (Driving reinvestment in research and development and responsible antibiotic use),^16^ that outlined the critical barriers for antibiotic research and development and helped reframe antibiotics as a global common pool resource, creating a solid economic rationale for public investment in antibiotic research and development to improve societal welfare.^27^^,^^28^ The result was broad acknowledgement that the conventional model for pharmaceutical research and development does not sufficiently incentivise antibiotic discovery and development, and emphasis on the balance between investment in push incentives, such as direct funding and grants, for basic science and preclinical research, and pull incentives, such as market entry rewards and subscription payments, to encourage investment in later clinical phases of development.

As a result, there has been significant public investment in push incentives for antibiotic research and development over the last decade. The EU established the New Drugs 4 Bad Bugs (ND4BB) programme with EUR 650 million between 2014 and 20 as part of a public-private partnership called the Innovative Medicines Initiative (IMI).^29^ ND4BB had multiple streams, including ENABLE and TRANSLOCATION that focused on drug discovery, COMBACTE that focused on drug development for gram-positive bacteria; and COMBACTE-CARE, COMBACTE-MAGNET, and IABC that focused on drug development for gram-negative bacteria.^30^ In 2018, the IMI AMR Accelerator was established to assist compounds through Phase II trials and has since invested almost EUR 500 million.^31^ Although, the majority of this funding is allocated to projects that target TB, with approximately EUR 35 million devoted to projects targeting Gram-negative bacteria.^31^

Several multilateral organizations have also been established with funding contributions from individual governments and non-governmental organisations (NGOs). A 2020 review of these initiatives,^32^ included Combating Antibiotic-Resistant Bacteria Biopharmaceutical Accelerator (CARB-X) with USD 500 million of funding between 2016 and 21 for preclinical research, the Global Antibiotic Research and Development Partnership (GARDP) with USD 270 million of funding between 2017 and 2023 for clinical research, and the Joint Programming Initiative on Antibiotic Resistance (JPIAMR) with EUR 234M of funding for novel therapeutics, and diagnostics (among other objectives) between 2012 and 24. There have also been private sector investments in push incentives, particularly for clinical stage trials, including USD 165 million of funding for the REPAIR programme between 2018 and 2023,^33^ and at least USD 1 billion of funding for the AMR Action Fund between 2020 and 2030.^34^

There has been relatively less investment in pull than on push incentives. Several approaches have been tried nationally in Europe (Table 1). In France, the HTA agency classifies medicines according to their added therapeutic value, from level I (major), via level II (important), level III (moderate), level IV (minor) to level V (non-existent).^35^ A referencing system exists to ensure that medicines classified as level I, II, & III are not reimbursed at prices lower than the lowest price across UK, Germany, Italy, and Spain. In 2015, this minimum price guarantee was extended to antibiotics classified as level IV.^36^ From 2021, this was also extended to antibiotics that are approved on the basis of non-inferiority clinical trials. In Germany, the definition of clinical benefit was amended to include resistance patterns, meaning antibiotics targeting resistant pathogens are not subject to internal reference pricing in which new drugs are assessed and priced relative to existing treatments for the same condition.^37^ In 2020, Germany also introduced the Act on Fair Competition among Statutory Health Insurance Funds (GKV-FKG),^38^ which speeds up the benefit assessment of the antibiotic targeting the abovementioned priority pathogens. This was successfully implemented in May 2022 with the antibiotic Cefiderocol (Fetcroja).^39^

**Table 1:** Summary of pull incentives for antibiotic research and development implemented in Europe.

Country	Name	Timeline	Mechanism type	Antibiotics targeted
France	Exception for antibiotics with ASMR level IV (minor), and those assessed by non-inferiority clinical trials	In effect since 2015	Medicines with’ moderate’ or higher added therapeutic benefit are guaranteed a price not lower than the lowest price across 4 reference countries. In 2015, this is extended to antibiotics with ‘minor’ added therapeutic benefit. In 2021, this is extended to antibiotics assessed by non-inferiority clinical trials	Antibiotics assessed as being ASMR level IV (minor), including those assessed by clinical non-inferiority trials
Germany	Changes in pharmaceutical legislation	In effect since 2017	*Ad hoc* exception of antimicrobials from internal price reference groups	Decided by reimbursement authority *ad hoc* taking into consideration resistance patterns
	Health Insurance Law	In effect since 2020	Automatic exception of ‘reserve’ antibiotics from internal price reference groups, accelerated reimbursement review process following EMA approval	‘Reserve’ antibiotics^a^
Sweden	Annual revenue guarantee	In effect since 2020	PHAS sets a minimum guaranteed annual revenue for selected originator antibiotics in exchange for a guaranteed supply volume. Payments are priced to secure access rather than incentivise research and development.	“Critical” pathogens in the WHO Priority Pathogens List
UK	Innovative models for the evaluation and purchase of antimicrobials	In effect since 2022	Annual fee, negotiated based on AMR-specific HTA, delinked from volume supplied	Pathogens on the WHO Priority Pathogens

Source: Adapted with permission from Gotham et al., 2021.^50^

AMR – antimicrobial resistance. ASMR – *amélioration du service médical rendu* (added therapeutic benefit). DRG – diagnosis-related group. EMA – European Medicines Agency. HTA – health technology assessment. PHAS – Public Health Agency of Sweden. PPL – priority pathogens list. QIDP – qualified infectious disease product. TLV – *Tandvårds-och läkemedelsförmånsverket* (Swedish Dental and Pharmaceutical Benefits Agency). ^a‘^Reserve group’ is to be defined by the Robert Koch Institute and the Federal Institute for Drugs and Medical Devices. There is also a separate definition of “reserve” antibiotics used by the WHO AWaRe (Access, Watch, Reserve) programme.

In England and Sweden, subscription-style payments (also known as revenue guarantees) have been used for reimbursement of antibiotics at the national level. Subscription payments can replace sales revenues (e.g., in the UK) with upfront payments each year for access to predetermined quantities of antibiotics or complement sales volumes (e.g., in Sweden), whereby an annual revenue guarantee is used in return to access to antibiotics. The National Institute for Health and Clinical Excellence (NICE) and NHS England implemented subscription payments to replace sales revenues for access to two new antibiotics as of April 2022, Cefiderocol (Fetcroja) and Ceftazidime/Avibactam (Zavicefta) for a total of £10 million a year for each antibiotic.^40^ The Swedish government uses an annual revenue guarantee of SEK4 million (approximately €400,000) per product to access new and older antibiotics in low volumes.^41^ In July 2020, access to five antibiotics was secured in this manner: four new ones, namely cefotlozane/tazobactam (Zerbaxa), imipenem/cilastin/relebactam (Recarbrio), Cefiderocol (Fetroja), and Meropenem/Vaborbactam (Vaborem), and one older antibiotic Fosfomycin.^41^ The UK subscription payment initiative is priced to secure access and provide an incentive for research and development, whereas the Swedish initiative is priced only to secure access. An evaluation of the Swedish programme concluded that the programme successfully improved access to new antibiotics,^42^ and an analysis of availability of new antibiotics in high-income countries found higher levels of access in Sweden, alongside the UK and the US, than in most other countries.^43^

Beyond Europe, several pull incentives have been implemented in the US. In 2012, the Generating Antibiotic Incentives Now (GAIN) Act was enacted allowing expedited regulatory assessment and an extension of market exclusivity of new antibiotics by five years.^44^ However, the evidence so far suggests that these incentives have not significantly influenced antibiotic research and development.^45^ In 2019, the Centres for Medicare and Medicaid Services (CMS) also added new antibiotics to eligibility criteria for “new technology add-on payments (NTAPs)” to attempt to increase access and uptake of new antibiotics.^46^ However, it has been emphasised NTAPs are unlikely to significantly impact revenues for antibiotic developers as eligible antibiotics are typically sold in small volumes.^47^ Over the last few years, the US Congress has been debating the Pioneering Antimicrobial Subscriptions to End Upsurging Resistance (PASTEUR) Act which would establish subscription-style contracts with antibiotic developers that bring new antibiotics that address unmet need to market.^48^ The scheme would have a total budget of US$6 billion over several years, but so far Congress has not passed the bill because of concerns regarding financial impact, lack of consensus on eligibility criteria for new antibiotics, and uncertainty whether rewards will consistently be provided to new antibiotics with added therapeutic benefit.^49^ Japan also plans to launch subscription payments for new antibiotics by April 2024,^50^ and Canada is also planning to implement subscription payments for antibiotics.^51^

Internationally, the combined use of push and pull incentives has contributed to several new antibiotics achieving regulatory approval over the last five years. According to 2022 WHO review of the antibiotic pipeline, there have been 12 new antibiotics that have received regulatory approval between July 2017 and November 2021.^4^ The WHO also assesses to what extent new antibiotics have innovative characteristics, including a known absence of cross-resistance with existing antibiotics; new chemical class; new target; and new mechanism of action. To date, almost no new antibiotics have innovative characteristics and are therefore vulnerable to cross-resistance with existing antibiotics. Moreover, only one new antibiotic targets the priority pathogens Carbapenem-resistant *A. baumannii*, and Carbapenem-resistant *P. aeruginosa*. The WHO describes the supply of recently approved new antibiotics, and those in clinical development, as “insufficient” to address the growing threat of antibiotic resistance.^4^ However, the WHO describes the preclinical pipeline as innovative, with many product types and projects widely distributed geographically.^4^ Therefore, there is an unmet need for additional push incentive funding to bring these pre-clinical products forward to the clinical pipeline, combined with investment in clinical trial networks and scientific expertise to coordinate and deliver the necessary clinical trials for antibiotic clinical development.

## Why is the antibiotic pipeline still not sufficient to tackle antimicrobial resistance?

This antibiotic pipeline remains insufficient to tackle AMR because of a complex range of scientific, economic, and regulatory factors that can be classified according to different stages of the drug development pathway.^15^ From a scientific perspective, several barriers exist. Despite sequencing the first complete bacterial genome completed as early as 1995, scientists have been largely unsuccessful in screening target genes to identify drug candidates for new antibiotics.^52^ Instead, developing novel chemical structures that inhibit validated cellular targets appears to be a more promising strategy for developing compounds capable of destroying resistant bacteria. However, discovering novel chemical structures safe for human consumption is very difficult. Moreover, destroying Gram-negative rather than Gram-positive bacteria has proven particularly challenging as Gram-negative bacteria have a protective outer membrane.^53^ This means that many antibiotic developers have focused on minor modifications to pre-existing classes of antibiotics. There is also limited technical and scientific expertise required to develop innovative solutions to these challenges in both academia and industry.^54^ In 2017, it was estimated there were only approximately 500 scientists active in antibiotic research and development.^55^ From a clinical perspective, the ongoing consolidation of clinical microbiology departments across Europe, and internationally, has also accelerated this decline in the number of scientists active in antibiotic research and development.^56^^,^^57^

Collectively, these scientific barriers mean that the rate of failure during preclinical and clinical phases for antibiotics under development is exceptionally high. The success rate of moving an antibiotic from the preclinical research stage to achieving regulatory approval has been estimated as low as between 1.5 and 3.5% and can take between 10 and 15 years.^58^ This high failure rate makes investments in antibiotic preclinical and early-stage clinical research a precarious proposition. Notably, a funding gap remains for the antibiotic candidates that reach these preclinical and early-stage clinical research stages (ominously referred to as the “valleys of death”). Here, many antibiotic candidates are abandoned because of a lack of investment. Investors also cannot be sure they will receive a significant return on investment if an antibiotic candidate successfully reaches the market, as launching and commercialising a new antibiotic is a financially risky prospect. New antibiotics have low sales volumes, are typically used as last-line options, and are often sold for low prices due to competition with pre-existing generics and their regulatory approval is based on data from non-inferior clinical trials.^59^

Many antibiotic developers that have launched antibiotics have subsequently experienced significant financial losses or filed for bankruptcy.^32^^,^^60^, ^61^, ^62^ The expected profitability of a drug development project can be expressed by the net present value (NPV), which is a sum of all expected revenues and costs of a project adjusted for the value of money over time and risk of failure.^19^^,^^63^ The NPV of an antibiotic development project has been estimated at −50 million USD compared to +720 million USD for a neurological drug and +1.15 billion USD for a musculoskeletal drug.^64^ As such, most pharmaceutical companies have withdrawn from investing in antibiotic research and development and the majority of antibiotic development is driven by small- and medium-sized enterprises (SMEs). In 2021, the Pew Trust reported that – of the 38 pharmaceutical companies with antibiotics in clinical development – only two rank among the top 50 pharmaceutical companies by sales.^65^ This is a significant challenge as SMEs often lack the financial resources and technical expertise to develop innovative new antibiotics. A review of funding applications for antibiotic drug discovery projects by SMEs and academia identified several scientific and technical shortcomings, such as a lack of understanding of toxicology standards, insufficient data, and weak scientific rationale for potential compounds.^66^ Supporting innovative antibiotic drug discovery inputs will require the input of “antibiotic pipeline coordinators” that can facilitate scientific, technical, regulatory and commercial advice to antibiotic SMEs and academia involved in antibiotic research and development.^67^ Pre-existing initiatives such as CARB-X and GARDP already undertake these functions at different stages of the antibiotic research and development pathway.^67^

From the regulatory perspective, procedural differences between national and regional drug regulatory agencies in approving novel antibiotics make global licensing time-consuming and costly, including differences in patient selection criteria, specification of statistical parameters, definitions of clinical endpoints, and rules regarding expedited approvals.^55^ Companies launching new antibiotics also need the technical capabilities to submit applications to all relevant regulatory agencies, which is particularly challenging for SMEs with limited human resources. This also results in inconsistent access to new antibiotics for many patients, as antibiotic developers may prioritise regulatory approval in one market over another. Evidence of this can be seen in Table 2, with several new antibiotics approved by the Food and Drug Administration (FDA) and not yet approved by the European Medicines Agency (EMA). Although there have been efforts to align regulatory processes between the FDA, EMA, and the Japanese Pharmaceuticals and Medical Devices Agency (PMDA) through the EMA-FDA parallel scientific advice process,^68^ and FDA-EMA-PMDA tripartite meetings on regulatory approaches for the evaluation of antibiotics.^69^

**Table 2:** Selected policy options for EU-level incentives for antibiotic research and development.

	Subscription-style payments^a^	Market entry reward	Transferable exclusivity extension	Milestone payments
Targets High-priority medical need	Payments can be greater for antibiotics that target high-priority medical need, and altered as real-world data is generated	Rewards can be greater for antibiotics that target high-priority medical need	Length of TEE can be greater for antibiotics that target high-priority medical need, but the value of TEEs are challenging to forecast	Milestone payments can be prioritised for antibiotics candidates that target high-priority medical need, although difficult to assess potential effectiveness and safety of antibiotic candidates in earlier stages of development
Supports Antibiotic sustainability	Continued payments can be conditional on stewardship requirements. Payments can replace revenues from unit sales, which removes incentives to oversell antibiotics	In case of a one-off reward, stewardship agreements could be challenging to enforce. Alternatively, rewards can be spread over five years.	Stewardship agreements are challenging to enforce as they are one-off rewards.	Milestone payments can include stewardship, patient access, and environmental health agreements but are challenging to enforce as payments made during multiple stages of development and ownership of intellectual property rights may change throughout the development. This can be addressed by attaching these contractual agreements to intellectual property, rather than antibiotic developers.
	If these incentives allow antibiotic developers to retain revenue from unit sales of antibiotics, incentives to oversell antibiotics remain.			
Promotes patient access	Continued payments can be conditional on access agreements Ongoing payments can also support the financial sustainability of the antibiotic developer	In case of a one-off reward, access agreements could be challenging to enforce. Alternatively, rewards can be spread over five years.	Can include access agreements, but they are challenging to enforce as they are one-off rewards.	
		There is a risk that antibiotic developers may subsequently become bankrupt if the monetary value of these incentives does not recoup antibiotic research and development costs or additional revenue is mismanaged.		
Protects environmental health	Continued payments can be conditional on manufacturing standards for environmental health	In case of a one-off reward, environmental health agreements could be challenging to enforce. Alternatively, rewards can be spread over five years.	Can include environmental health agreements, but they are challenging to enforce as they are one-off rewards.	
Improves NPV	Improvements in expected NPV are dependent upon what extent payments or rewards reflect antibiotic research and development costs and risk of failure during each stage of development		Likely to provide a substantial size of incentive and significantly improve expected NPV	Increases NPV by reducing the risk of investments in antibiotic development
Enables SME participation	If the size of the incentive is large, then likely to make attracting investment by SMEs at early stages of development more feasible.			Supports SMEs at multiple stages of development. Payments can be targeted to funding gaps, including preclinical and early clinical development
Operational feasibility at the EU level	Proportional payments or contributions by member states to payments or rewards would need to be coordinated by a responsible institution such as the EIB, or HERA		Straightforward to implement at EU level from a regulatory perspective. Many member states are opposed to the use of TEEs	Milestone payments would need to reflect the costs of subsequent stages of research and development Proportional contributions by member states to milestone payments would need to be coordinated by a responsible institution such as the EIB or HERA.
	Responsibility for assessing antibiotic candidates and issuing payments or exclusivity extensions must be designated to a scientific committee or antibiotic pipeline coordinator at the EU level. Roles and responsibilities for monitoring compliance with antibiotic sustainability, patient access, and environmental health standards by antibiotic developers would also need to be designated to relevant institutions at the EU level.			
Financial feasibility at the EU level	Impact on EU budgets dependent upon the extent the European Commission shares the financial burden with member states to contribute to subscription payments or rewards		Straightforward to implement as negligible impact on EU budgets	Impact on EU budgets will be dependent upon the extent the European Commission shares the financial burden with member states to contribute to milestone payments
Financial feasibility at national level	The financial burden on member states could be spread over multiple years	If multiple rewards are granted over a short time, they could create a significant short-term financial burden on member states.	The uncertain financial impact on member states, but potentially responsible for high additional costs Auction process could be introduced to limit length of TEEs and financial impact	The number and size of milestones payments or investments by member states could be limited to ensure financial feasibility

a Subscription style payments are also known as annual revenue guarantees.

## What principles are needed for a holistic incentive package for antibiotic research and development?

There is consensus that no single incentive will be sufficient to stimulate antibiotic research and development, and a combination of push and pull incentives will be required. Each incentive has benefits and disadvantages regarding the expected impact and operational feasibility, and there are a number of principles that policymakers should consider when assessing different options for incentives.

First, a holistic incentive package for antibiotic research and development should reinforce broader public health objectives. In 2019, around 80% of the global health burden attributed to antibiotic resistance globally resulted from respiratory tract infections, bloodstream infections, and intra-abdominal infections.^70^ There is also a notable absence of effective antibiotics for resistant infections in children, infants, and neonates, despite the high rates of mortality and morbidity associated with resistant infections in these patient groups.^71^^,^^72^ Incentives could be designed that reward antibiotic developers proportionally according to what extent the global health burden of antibiotic-resistant bacterial infections is reduced to target innovation towards key clinical indications. Promoting antibiotic sustainability involves ensuring new antibiotics remain effective by limiting the emergence of resistant bacteria. This requires appropriate antibiotic stewardship. However, the traditional volume-based business model for medicines encourages pharmaceutical companies to increase sales to maximise return on investment before patent expiry. It is also important that patients can access new antibiotics, as prolonged infections and use of suboptimal broad-spectrum antibiotics can also drive resistance development.^73^ However, as launching new antibiotics in different markets can be financially risky, many new antibiotics are not launched in all countries.^43^ Finally, alongside terms and conditions for antibiotic stewardship reimbursement mechanisms for antibiotics need to include terms and conditions that ensure manufacturers limit the exposure of antibiotics to the environment.^74^ This also involves procurement of reaction intermediates and active pharmaceutical ingredients (APIs) from suppliers that commit to internationally agreed standards for antibiotic levels in manufacturing effluent.^75^

Second, any package of incentives for antibiotic research and development needs to ensure the expected return on investment increases. As mentioned above, the average NPV for antibiotic drug development is estimated to be negative.^76^ Launching antibiotics can be financially risky, with many developers declaring bankruptcy or experiencing significant economic losses.^32^^,^^60^, ^61^, ^62^ Improving the profitability of investments in antibiotic research and development will require designing incentives that reduce the costs or risk of investments in research and development and increase revenues from new antibiotics. Crucially, investments will need to be fully accessible by SMEs, as well as larger organisations such as CARB-X and GARDP, that drive innovation in antibiotic research and development. This will involve providing financial and technical support during preclinical and early-stage clinical development because SMEs have significantly smaller capital reserves than large pharmaceutical companies and cannot fund the necessary costs of preclinical studies (estimated between USD23.5–26.5 million in 2021) and Phase I trials (estimated between USD19.8–28.8 million in 2021).^58^

Third, it is important to consider the political, regulatory, legislative, industry and financial hurdles potentially faced during implementation of any incentives for antibiotic research and development. In Europe, there are particular challenges in navigating the complexities of EU legislation. For example, the boundaries between the scope of EU- and national-level health policies sometimes create tensions, as pre-existing treaties, such as the Treaty on the Functioning of the European Union, state the EU must respect member states’ autonomy in operating their health systems.^77^ Different incentives also have varying financial implications for the European Commission and member states, depending upon what extent the European Commission shares the burden with member states of financially contributing to different incentives. For incentives implemented at the EU level but financed by member states, there would need to be an institution, such as the European Investment Bank (EIB) or the Health Emergency Preparedness and Response Authority (HERA), responsible for coordinating or collecting proportional financial contributions. There would also need to be collective agreement among member states on size of incentives, and what metric proportional payments are determined (for example GDP). Governance of the incentive package is also essential, as there needs to be transparency regarding the methodological assumptions and rationale behind the design (including the size of incentives) and implementation at different stages of the antibiotic research and development pathway. This will involve broad consultations with patients, health professionals, industry, academic and government stakeholders and representatives. The societal value and likely success of alternative antibiotic candidates must also be modelled to ensure investments are optimally distributed. The aim should be to achieve an end-to-end approach to antibiotic research and development, with full oversight of where additional investment or technical and scientific support is required. Achieving this will require designating responsibility for assessing antibiotic candidates to a scientific committee or an “antibiotic pipeline coordinator”.^67^ Such a body could also collaborate with other institutions such as the WHO, European Centre for Disease Prevention and Control (ECDC), and the Africa Centres for Disease Control and Prevention (CDC) to monitor resistance patterns and prospective identify which clinical indications antibiotic developers should target to maximise public health benefits.

## How suitable are policy options currently being considered at the EU level?

There is consensus among EU policy makers that increased investment is needed in new and pre-existing incentives to revitalise the antibiotic pipeline.^10^^,^^16^^,^^24^ The purpose of the following section is to critically appraise different incentive options for antibiotic research and development using the aforementioned principles for holistic incentive package for antibiotic research and development. The selection criteria for incentives analysed were those that have been proposed by the European Commission, and member states. The European Commission has proposed using transferable exclusivity extensions to incentivise research and development of new antibiotics within planned revisions to the EU pharmaceutical legislation.^6^ Whereas, fourteen member states have also recently suggested using subscription payments, market entry rewards (MERs), and milestone payments as alternatives.^10^ These incentive options were also included as options in recently published Council of the European Union recommendations on AMR to be included when developing a voluntary multi-country pull incentive scheme. The results of our analysis are summarised narratively within Table 2.

During the development of this article, the Directorate General for Health Emergency Preparedness and Response Authority (HERA) published a report that modelled the expected cost and impact on expected NPV different stages of drug development for subscription payments, MERs, and milestone payments.^78^ We also reference the main findings of the HERA report in relation to the expected cost and impact of expected NPV at the beginning of phase 1 clinical trials for each incentive.^78^ We focus on the beginning of phase 1 clinical trials as this is the development stage when many antibiotic developers struggle to source funding,^65^ although the full results on expected NPV at all stages of development are contained within the report.^78^

### Subscription payments

Subscription payments (also known as revenue guarantees) for antibiotics either pay developers an annual fixed price to access antibiotics upfront (for example in the UK) or guarantee annual revenues (for example in Sweden).^79^ The Joint Action on Antimicrobial Resistance and Healthcare-Associated Infections (JAMRAI) has proposed a mechanism for subscription payments through an annual revenue guarantee, modelled on the Swedish approach, which could be implemented at the EU level.^26^ Participation among member states would be optional, although ideally the goal should be full participation by >90% of the EU population (or GDP). The EC would be responsible for coordinating tenders for new antibiotics and ensuring national financial responsibility for the guarantee would be apportioned and agreed upon. The size of subscription payments could vary proportionally to what extent new antibiotics target priority pathogens or clinical indications with a significant health burden. While antibiotic developers would retain sales revenues above and beyond the annual revenue guarantee if the annual revenue guarantee were set at a sufficient level, this should reduce incentives to oversell antibiotics, which can harm stewardship efforts (i.e. antibiotic sustainability). An alternative approach, used in the UK, is when subscription payments replace revenue from unit sales which removes incentives to oversell antibiotics. Tender agreements would include access requirements to provide antibiotics and could include conditions on environmental health manufacturing standards.

As subscription payment models are typically agreed upon towards the end of antibiotic development, the impact of this incentive on stimulating the antibiotic pipeline would depend on the size of incentive and whether the institution responsible for assessing antibiotic candidates provides reliable signals to antibiotic developers during the early stages of development that they may be eligible for subscription payments upon market approval (through a mechanism of “pre-qualification”). The same applies to other incentives implemented towards the end of antibiotic development, including MERs and TEEs. The HERA study modelled the impact of two options for revenue guarantees of €150 million and €100 million per year, that would complement revenues generated from sales, and expected to cost €750 million and €421 million over 10 years for each new antibiotic.^78^ According to the modelling within the report, the expected NPV would become positive at the start of phase 1 clinical trials for around 50% of projects if the first option was implemented, and for around 25% of projects if the second option was implemented.^78^

Implementation feasibility at the EU level may depend upon achieving consensus among member states regarding the size of payments, the public health value of the new antibiotic, and sales volumes required to meet demand. The HERA study conducted a survey of member states regarding their interest in participating in the mechanism for subscription payments proposed by JAMRA, and 80% of member states responded stating they needed more information before taking a position.^78^

### Market entry rewards (MERs)

MERs are a financial reward for antibiotic developers that successfully achieve regulatory approval for a new antibiotic. They could be a one-off reward, or payments could be spread over several years. They may also be granted in exchange for intellectual property rights to allow earlier entry of generic manufacturers and diversification of supply. The size of MER could vary according to the extent to which a new antibiotic targets high-priority medical needs. MERs can include stewardship, access, and environmental health agreements, although these may be challenging to enforce if the MER is a one-off payment. There is also a risk that antibiotic developers may later become bankrupt if the size of MER does not reflect research and development costs or if funds are mismanaged. MERs would likely benefit both large pharmaceutical companies and SMEs since they would make attracting investment by SMEs at early stages of development more feasible if appropriately sized.

MERs can be used to fund post-approval costs related to pharmacovigilance or commercialisation,^80^ which is essential as these costs have contributed to several antibiotic developers either becoming bankrupt or experiencing significant economic losses.^81^ Operationally, they would be feasible to implement at the EU level as they are a one-off reward, which could be granted following regulatory approval by the EMA. However, if funded by member states rather than the European Commission, collecting proportional payments to finance MERs may be challenging. If multiple MERs were granted simultaneously, this could be responsible for high short-term costs. In contrast, the financial burden of subscription payments could be spread over multiple years. However, it should be noted that the costs of MERs can be spread over multiple years. For example, DRIVE-AB recommended that the financial costs of MERs should be spread over up to five years.^16^ The HERA study assumed MERs would be paid over six years, with higher payments in the first two years followed by a smaller revenue guarantee that would complement revenues from sales for four years.^78^ The HERA study modelled three combinations; the first option involved payments of €500 million for two years followed by a revenue guarantee of €125 million, the second option involved payments of €330 million for two years followed by a revenue guarantee of €85 million, and the third option involved payments of €250 million for two years followed by a revenue guarantee of €50 million. According to the modelling within the report, the expected NPV would become positive at the start of phase 1 clinical trials for around 75% of projects if the first option was implemented, for around 50% of projects if the second option was implemented and for around 25% of projects if the third option was implemented.^78^

### Transferable exclusivity extensions (TEEs)

Transferable exclusivity extensions (TEEs) are a reward granted to antibiotic developers that successfully develop and launch a new antibiotic that can be used to extend the intellectual property rights of another medicine for a fixed period (i.e., up to 12 months).^82^^,^^83^ They can be used by the antibiotic developer or auctioned for the highest price. Advantages of TEEs emphasised by the pharmaceutical industry include ease of implementation, no requirement for upfront government funding, and benefits for pharmaceutical companies of all sizes.^84^^,^^85^ TEEs would also provide a substantial size of incentive as they would be used for expensive, high-selling brand-name drugs. As a result, the would also be responsible for substantial additional costs for national healthcare systems. The exact financial impact of TEEs is difficult to establish, as estimates are sensitive to the number granted, which drugs are nearing patent expiry and the impact of generic market entry on prices. Årdal et al., 2020 estimate the costs to European healthcare systems for a single TEE could be over €3 billion,^17^ whereas other industry-sponsored analyses suggest it could be less than €1 billion.^86^^,^^87^ There are also important ethical implications to consider as TEEs would increase the financial burden for national health systems in another therapeutic area, and potentially delay more widespread access to medicines that often follows generic market entry.^88^ TEEs are also a one-off reward, making it difficult to include strict conditions to ensure access to specific markets or to limit environmental exposure to antibiotics during manufacturing. This is a particular challenge if the TEE is granted and the antibiotic developer subsequently declares bankruptcy. While the length of TEE can vary to link the public health value of new antibiotics to the size of reward received, this is challenging in practice because the value of a TEE is determined by which expensive, high-selling brand-name medicines are nearing patent expiry when granted, rather than whether an antibiotic addresses high-priority medical needs. One approach suggested to mitigate against potential high costs of TEEs is to auction TEEs based upon length of exclusivity extension, as pharmaceutical companies with more expensive and high-selling medicines would bid using shorter exclusivity extensions with the knowledge they can still secure substantial additional revenues.^89^

### Milestone payments

Milestone payments involves granting funding to developers at specific stages of antibiotic development. Milestone payments could target high-priority medical needs by establishing eligibility criteria, but this would be operationally challenging and require substantial technical expertise. This function could be undertaken by an “antibiotic pipeline coordinator”, who should have oversight of the antibiotic pipeline internationally and facilitate cooperation and exchange of technical expertise among relevant stakeholders across the antibiotic pipeline. Such an antibiotic coordinator would also be tasked with interpreting the reliability, validity, and transparency of clinical trial data. Some institutions already undertake this role at different stages of development, including CARB-X and GARDP.^67^ Milestone payments could be used to support SMEs which struggle to secure funding during the preclinical and early stages of clinical development. Milestone payments can include stewardship, access, and environmental health agreements but these are challenging to enforce as milestone payments could be made during multiple stages of development and ownership of intellectual property rights may change. This can be addressed by attaching these contractual agreements to intellectual property, rather than antibiotic developers. Another drawback of milestone payments is that there would be no guarantee that antibiotic candidates would successfully achieve market approval, whereas other incentives (e.g., subscription payments, MERs, or TEEs) would only be granted once market approval was achieved. Baraldi et al., 2019 modelled milestone payments and concluded they would be most effectively deployed following the completion of phase 1 clinical trials.^90^ The HERA study modelled the impact of granting a €60 million milestone payment following the completion of phase 1 clinical trials, and concluded that the expected NPV of around 25% of projects starting phase 1 clinical trials would become positive if this was implemented.^78^ The modelled estimated the expected costs of milestone payments for each new antibiotic would be €169 million, because multiple milestone payments would need to be granted to bring a new antibiotic to market.

### The need for continued focus on push incentives by the EU

While the policy discussion at the EU level is predominantly focused on pull incentives,^10^ it is vital to emphasise the continued need for push incentives to encourage antibiotic research and development for drug discovery science, preclinical, and early clinical research. This was also recently acknowledged as a priority within the recently published Council of the European Union recommendations on AMR.^9^ This is important because the high failure rate in earlier development stages means that the drug discovery and preclinical pipeline will not be sustainable without some form of public funding or subsidisation. There are strong economic arguments for public subsidisation in this therapeutic area, as having a supply of effective antibiotics and reduced infection can be considered a “public good” that benefits society holistically.^91^ With this in mind, the EU needs to consider balancing push and pull incentives together to improve the quality of the antibiotic pipeline. Completing the ND4BB programme (including ENABLE and TRANSLOCATION programmes focused on drug discovery) means an unmet need for continued push incentive funding by the EU focused on drug discovery and preclinical research. While ENABLE has continued within the ENABLE-2 programme, this was launched using limited funding from the Swedish government and operates on a much smaller scale.^92^ The establishment of the Innovative Health Initiative (IHI) (a budget of €2.4 billion between 2021 and 27),^31^ and the Health Emergency Response Authority (HERA) (with a budget of approximately €6 billion for the years 2021–2027) represent significant opportunities to reinvest in push incentives,^7^ although their investment properties are yet to be defined.

## Conclusion: advocating for a global solution to reinvigorate the antibiotic development pipeline

Over the last two decades, the EU has demonstrated global leadership in combating AMR through interventions such as enhanced surveillance, strengthened legislation of antimicrobial use, advocating for a “One-Health” approach, and investment in push incentives for antibiotic development.^12^ Despite progress, rates of AMR continue to grow, and the antibiotic development pipeline remains insufficient to meet public health needs. As policy debate regarding the optimal deployment of push and pull incentives intensifies internationally, the EU must advocate for urgent and coordinated action at the global level through platforms such as the WHO, and G7 and G20 group of countries. In practical terms, this requires building consensus regarding the appropriate size of incentives, updating target drug profiles and clinical indications, creating platforms to share technical and scientific expertise, and avoiding duplication of efforts. The EU and other international partners must also acknowledge the global nature of AMR and consider how to support initiatives that aim to secure sustainable access to new and pre-existing antibiotics in LMICs. This is outlined as a priority within the 2022 EU Global Health Strategy.^8^ and many policy options warrant further examination such as joint procurement mechanisms, license and technology transfer agreements, and strengthening manufacturing capacity in LMICs. There are also opportunities for the EU to advocate for a more prominent role for the WHO in international governance, and to leverage the European and Developing Countries Clinical Trials Partnership (EDCTP) as a platform for clinical development of new antibiotics.

## Contributors

MA drafted the manuscript. MA and EM designed the analysis. All other authors provided comments and edits to iterative versions of the manuscript.

## Declaration of interests

The authors have no relevant conflicts of interest to declare.
